# Adult-Onset Neuronal Intranuclear Inclusion Disease Initially Manifesting as Bladder Dysfunction: A Case Report

**DOI:** 10.7759/cureus.105488

**Published:** 2026-03-19

**Authors:** Anna Yamaki, Hirofumi Sekino, Satoshi Kawana, Ryo Yamakuni, Shiro Ishii, Hiroshi Ito

**Affiliations:** 1 Department of Radiology and Nuclear Medicine, Fukushima Medical University, Fukushima, JPN; 2 Department of Diagnostic Pathology, Fukushima Medical University, Fukushima, JPN

**Keywords:** autonomic dysfunction, bladder dysfunction, diffusion-weighted mri, neuronal intranuclear inclusion disease, notch2nlc

## Abstract

Neuronal intranuclear inclusion disease (NIID) is a rare neurodegenerative disorder characterized by eosinophilic intranuclear inclusions and diverse clinical manifestations. In adults, NIID typically manifests as cognitive impairment, while autonomic dysfunction, such as bladder issues, is uncommon as an initial manifestation. We present a case of adult-onset NIID where bladder dysfunction preceded cognitive impairment by several years. A woman in her sixties experienced progressive urinary incontinence and voiding difficulties, necessitating intermittent self-catheterization. Early brain magnetic resonance imaging (MRI) revealed ribbonlike hyperintensity at the frontal corticomedullary junction on diffusion-weighted imaging, along with multifocal white matter lesions. However, no cognitive impairment was evident at that time. Subsequent cognitive decline progressed gradually. Further evaluation, including skin biopsy, showed ubiquitin-positive intranuclear inclusions, and genetic testing identified an abnormal guanine-guanine-cytosine (GGC) repeat expansion in the NOTCH2NLC gene, confirming the diagnosis of NIID. This case highlights that bladder dysfunction could precede cognitive impairment by years in adult-onset NIID. NIID should be considered in unexplained bladder dysfunction cases, particularly with suggestive MRI findings. Early recognition and minimally invasive skin biopsies can aid in prompt diagnosis.

## Introduction

Neuronal intranuclear inclusion disease (NIID) is a rare degenerative disorder characterized by eosinophilic intranuclear inclusions throughout the body [1,2]. A wide range of clinical manifestations, primarily neurological symptoms, are observed in both sporadic and familial cases [1,3]. Historically, NIID diagnosis relied on rectal biopsy, nerve biopsy, or autopsy [1,2]. However, skin biopsy has emerged as a valuable diagnostic tool, enhancing accessibility and contributing to an upsurge in reported cases [4]. Furthermore, an expanded guanine-guanine-cytosine (GGC) repeat in the NOTCH2NLC gene on chromosome 1 has been identified as the underlying cause of NIID, facilitating genetic diagnosis [3].

NIID is categorized into infantile, juvenile, and adult-onset forms depending on the age at onset [1,2]. The adult-onset variant primarily manifests cognitive impairment as the core symptom, along with peripheral neuropathy, autonomic dysfunction, and involuntary movement [1,3]. Autonomic dysfunction encompasses orthostatic hypotension and bladder dysfunction, with the latter showing various symptoms such as urinary frequency, incontinence, and voiding difficulties [1,3,5]. A Korean cohort study indicated that 48% of patients with NIID experienced bladder dysfunction [3], although it was an uncommon initial symptom. This report describes a case of adult-onset NIID in which bladder dysfunction preceded cognitive impairment, with the diagnosis confirmed seven years after symptom onset.

## Case presentation

The patient, a woman in her sixties, had a history of surgery for a cerebellopontine angle tumor (epidermoid cyst) and bilateral breast cancer. The patient had no significant family history except for her father's death from cerebral infarction.

She had been experiencing urinary incontinence for three years and had noticed voiding difficulties in the past year. As her symptoms deteriorated, she resorted to abdominal straining and manual pressure to urinate. Subsequently, she sought care at a nearby urology clinic, where she was then referred to our urology department for further evaluation. Following this, she underwent intermittent self-catheterization. Because the urological evaluation did not identify a local cause and neurogenic bladder was suspected, the patient was referred to the Department of Neurology, where a brain MRI scan was subsequently performed. At the initial visit, she was independent in activities of daily living. Nerve conduction studies demonstrated reduced motor conduction velocities in the median, ulnar, and tibial nerves; reduced sensory conduction velocities in the median, ulnar, and sural nerves; and prolonged F-wave latencies in the median and ulnar nerves. There was no significant muscle weakness on neurological examination.

**Figure 1:**
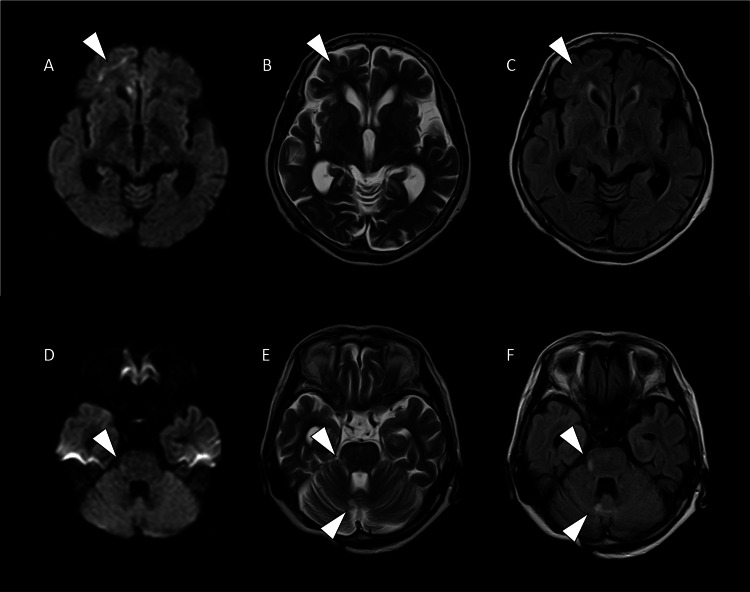
Brain MRI at initial presentation DWI: diffusion-weighted imaging; T2WI: T2-weighted imaging; FLAIR: fluid attenuated inversion recovery DWI (A,D) showing a ribbonlike hyperintensity (arrowhead) at the right frontal corticomedullary junction and hyperintense lesions (arrowhead) in the middle cerebellar peduncle. T2WI (B,E) and FLAIR (C,F) images showing hyperintense lesions (arrowhead) in the right frontal corticomedullary junction, middle cerebellar peduncle, and paravermian cerebellar region

Diffusion-weighted imaging (DWI, b = 1000), T2-weighted imaging (T2WI), and fluid attenuated inversion recovery (FLAIR) sequences revealed ribbonlike, high-intensity signals at the right frontal corticomedullary junction. Additionally, high-intensity lesions were noted in the middle cerebellar peduncle and paravermian regions on T2WI and FLAIR images (Figure 1). These lesions did not show corresponding low signal intensity on apparent diffusion coefficient (ADC) maps. However, the relationship between multifocal brain lesions and neurogenic bladder remains unclear. Possible explanations include postoperative changes following cerebellar epidermoid tumor surgery or chemotherapy-related effects of breast cancer treatment. Consequently, the patient was placed under observation, and the neurological examination performed at the initial visit revealed no abnormalities.

Two years after the initial visit, the patient developed subjective forgetfulness, which gradually progressed over the next two years. Subsequently, she sought evaluation at a local neurosurgery clinic, where NIID was suspected based on the MRI findings, leading to a referral to our neurology department. A neurological examination revealed sensory impairment in the right facial area and the upper and lower extremities on the right side.

**Figure 2:**
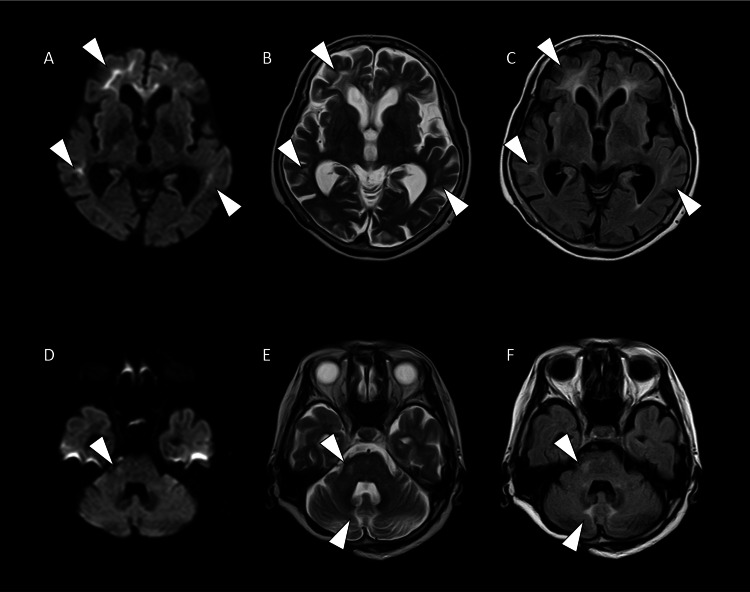
Brain MRI at diagnosis DWI: diffusion-weighted imaging; T2WI: T2-weighted imaging; FLAIR: fluid attenuated inversion recovery DWI (A,D) showing a ribbonlike hyperintensity (arrowhead) at the right frontal and temporal corticomedullary junctions, along with a hyperintense lesion (arrowhead) in the middle cerebellar peduncle. T2WI (B,E) and FLAIR (C,F) images showing hyperintense lesions (arrowhead) in the right frontal and temporal corticomedullary junctions, middle cerebellar peduncle, and paravermian cerebellar region. Compared with the images obtained at the initial visit (four years earlier), these abnormalities were more conspicuous

Subsequent MRI scans showed continued and more distinct delineation of high-intensity lesions on DWI, T2WI, and FLAIR at the right frontal and temporal corticomedullary junctions, the middle cerebellar peduncle, and the paravermian cerebellar region. These findings were accompanied by an increase in white matter lesions and generalized brain atrophy (Figure 2). The imaging findings and symptoms of peripheral neuropathy were compatible with those of NIID, and NIID was suspected.

**Figure 3:**
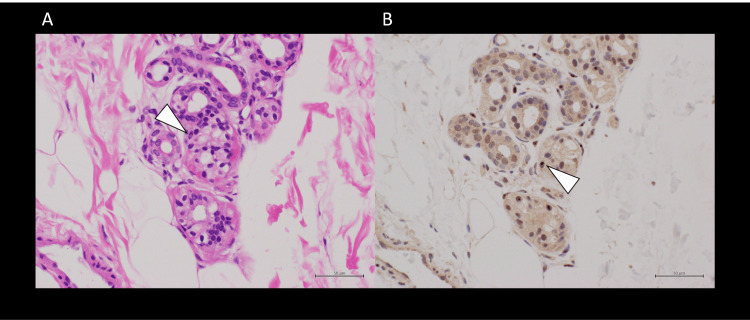
Histopathological and immunohistochemical images of the skin biopsy Hematoxylin and eosin staining (A) revealed intranuclear inclusions (arrowhead) in sweat gland epithelial cells. The intranuclear inclusions (arrowhead) of sweat gland epithelial cells identified on hematoxylin and eosin staining showed strong immunopositivity for ubiquitin (B)

Skin biopsy revealed numerous intranuclear inclusions that were positive for ubiquitin immunostaining and hematoxylin and eosin (H&E) staining in sweat gland epithelial cells, dermal fibroblasts, adipocytes, and smooth muscle cells of the vascular media (Figure 3). Genetic testing revealed an abnormal expansion of the GGC repeat in the NOTCH2NLC gene. Based on these findings, the patient was diagnosed.

After being discharged, the patient regularly attended our neurology clinic. Memantine was prescribed to address cognitive impairment. A urethral catheter was placed to manage bladder dysfunction five years post-initial visit. Over time, cognitive impairment, gait instability, and autonomic symptoms gradually progressed. Seven years after her initial presentation, the patient became reliant on external support for independent living.

## Discussion

This case illustrates NIID in which bladder dysfunction was the initial manifestation. NIID is a neurodegenerative disorder characterized by eosinophilic hyaline-like intranuclear inclusions in the central nervous system, peripheral nerves, and visceral organs [1]. Clinical presentations vary and include pyramidal tract signs, cerebellar ataxia, dementia, and autonomic dysfunction [1]. Cognitive impairment is the most frequently reported initial symptom in adult-onset cases, followed by miosis, ataxia, and impaired consciousness. However, autonomic dysfunction, such as miosis and bladder dysfunction, is also frequently noted [1]. Cognitive impairment is a common manifestation of NIID. Previous studies have reported progressive cognitive decline in approximately 64% [3] of patients, and dementia-dominant phenotypes have been described in adult-onset NIID.

Historically, most cases have been considered sporadic [1]. The identification of the GGC repeat expansion in the NOTCH2NLC gene as the causative mutation led to the recognition of familial NIID with autosomal dominant inheritance [3]. Earlier clinical diagnoses suggested that approximately 70% of cases were sporadic [1], but recent studies incorporating genetic testing have revealed a higher prevalence of familial cases [3]. Symptom patterns, broadly similar in sporadic and familial NIID, can be categorized as dementia-predominant and limb-weakness-predominant types [1]. The dementia-predominant type typically emerges after 40 years of age, characterized primarily by cognitive impairment, with some patients also showing autonomic symptoms like miosis or bladder dysfunction [1]. The limb-weakness-predominant type, affecting individuals under 40 years, is characterized by muscle weakness, often with sensory disturbances, miosis, and bladder dysfunction [1]. Bladder dysfunction occurs in approximately 30% of sporadic cases and approximately 60% of familial cases [1]. However, cases such as ours, where bladder dysfunction is the initial symptom despite late-onset disease, are rare, though several similar cases have been documented [4,6]. Therefore, NIID should be considered in patients with unexplained bladder dysfunction as a potential differential diagnosis [4,6].

A characteristic brain MRI finding in individuals with NIID is the persistent hyperintensity along the corticomedullary junction in the frontal region on DWI [1,7]. Lesions in the cerebral white matter were evident on T2-weighted and FLAIR sequences [1,7]. Furthermore, hyperintense lesions could manifest in the brainstem (midbrain and pons), paravermian area, and middle cerebellar peduncles on DWI, T2-weighted, and FLAIR images [1,3]. Although the characteristic high-intensity signal along the corticomedullary junction on DWI strongly suggests NIID, similar diffusion patterns have been reported in fragile X-associated tremor/ataxia syndrome (FXTAS), and genetic testing is essential when differentiation remains uncertain [8,9]. In addition, seizure-induced signal changes, toxic leukoencephalopathies, inherited leukodystrophies, and other neurodegenerative diseases should be considered in the differential diagnosis depending on the clinical context [8].

In the present case, while conventional cognitive impairment was not evident initially, brain MRI revealed distinctive NIID features early on. Despite bladder dysfunction being an uncommon initial symptom, it is frequently reported in sporadic adult-onset NIID and was present early in this case [1]. NIID should be actively suspected even if the initial symptoms are atypical, given the presence of these characteristic imaging findings. Since skin biopsy is minimally invasive and aids in confirming a definitive diagnosis, it should be promptly included in the diagnostic evaluation [1,7].

## Conclusions

In cases of adult-onset NIID, bladder dysfunction might precede cognitive impairment, serving as the primary symptom. Brain MRI is valuable for diagnosis, because characteristic imaging findings may be present at an early stage. Skin biopsy should be considered if typical imaging findings are noted, even in cases of atypical symptoms such as isolated bladder dysfunction without cognitive impairment.
